# A System for Sentiment Analysis of Colloquial Arabic Using Human Computation

**DOI:** 10.1155/2014/631394

**Published:** 2014-04-29

**Authors:** Afnan S. Al-Subaihin, Hend S. Al-Khalifa

**Affiliations:** Information Technology Department, College of Computer and Information Sciences, King Saud University, Riyadh 11496, Saudi Arabia

## Abstract

We present the implementation and evaluation of a sentiment analysis system that is conducted over Arabic text with evaluative content. Our system is broken into two different components. The first component is a game that enables users to annotate large corpuses of text in a fun manner. The game produces necessary linguistic resources that will be used by the second component which is the sentimental analyzer. Two different algorithms have been designed to employ these linguistic resources to analyze text and classify it according to its sentimental polarity. The first approach is using sentimental tag patterns, which reached a precision level of 56.14%. The second approach is the sentimental majority approach which relies on calculating the number of negative and positive phrases in the sentence and classifying the sentence according to the dominant polarity. The results after evaluating the system for the first sentimental majority approach yielded the highest accuracy level reached by our system which is 60.5% while the second variation scored an accuracy of 60.32%.

## 1. Introduction

The task of sentiment analysis is known to involve many disciplines. Such multidisciplinary nature is common to many computational tasks that seek to solve problems dealing with the semantics of human-generated content (a.k.a. user generated content). Sentiment analysis aims to automatically identify sentences with evaluative meaning and classify them according to their polarity, as either negative, positive, or neutral. Such task may appear simple in concept; however, when attempting to implement a tolerably accurate tool, such a tool would require the collaboration of several disciplinary fields including psychology, linguistics, and computer science. In addition to these fields, we introduce the application of human-based computing approaches to the problem of sentiment analysis.

Human computation is defined as the area of harnessing human knowledge and processing power to solve problems that computers are unable to solve yet. The vision of human computing applications is to design computing systems that systematically integrate human input as well as computer computing output in order to facilitate solving problems that neither one of them is able to solve on their own [1]. The great popularity of this new field is attributed to the ease of integrating human collective intelligence at a costly manner using the World Wide Web. By providing proper incentives, human power can be channeled and gathered in a feasible way.

The aim of this paper is to design and implement a system that can extract the sentiments of Arabic content found in Arabic websites that has informal nature. Our novel technique merges the area of human-based computing which states that some steps of a system can be outsourced to human participation with the area of natural language processing. Additionally, to enhance our system with dynamically and periodically updated resources, our system has two different components that operate independently and in parallel: the game component and the sentiment analyzer. The output of one component is used as an assistive resource for the other component.

Therefore, this paper is structured as follows.  Section 2 capitalizes on the significance of our study.  Section 3 provides some related work in the field of sentiment analysis and opinion mining techniques for Arabic text.  Section 4 describes our system design and implementation.  Section 5 presents the results of our system evaluations.  Section 6 discusses our system results and limitations. Finally, Section 7 concludes the paper with final remarks.

## 2. Significance of the Study

There are several approaches to analyze English text on the Internet and provide user sentiments and opinions towards a certain subject. Mainly, these approaches are divided into two categories: supervised and unsupervised techniques. The majority of supervised approaches investigate applying machine learning algorithms over natural language processing and information retrieval applications. Unsupervised approaches devised applying classification algorithms that are assisted by language lexicons and dictionaries. Many tools were developed and made useful to English users. However, the area of Arabic sentiment analysis of information contained on the web is not well established.

A specific challenge is encountered when attempting to deal with Arabic content found in Arabic informal websites. Arabic content in these websites is generally characterized to be written in a highly informal Arabic language that is used in colloquial speaking. This language is subject to differences in dialects of Arabic regions and difficult to model and analyze. An additional characteristic is that expressions used by web users are highly subject to trends that give different meanings and usages to Arabic evaluative phrases over time. This makes the task of creating a timeless tool difficult. Upon conducting a specific study to identify the specific features of Arabic evaluative language found in Arabic websites, we propose the usage of a human-based computing to solve such problem.

## 3. Related Work

Related work in the field of sentiment analysis and opinion mining, in addition to work relating to the applications and techniques of human-based computing, will be reviewed and discussed in this section.

Firstly, a brief and introductory review is presented on the research pertaining to subjectivity analysis which is the detection of opinionated content. The following is a survey of the efforts to conduct sentiment analysis and opinion mining to different types of data and domains. Afterwards, we will review the area of human-based computing and its used techniques and common applications.

While researching the literature for endeavors to apply sentiment analysis and opinion mining techniques over Arabic text, we have detected a relatively low volume of research. The approaches we have found for Arabic language had various techniques to apply them over Arabic text. Some simply adopt techniques that have been proposed for other languages while others, albeit a rare endeavor, devised new techniques for Arabic.

In 2006, a new approach was proposed to conduct sentiment analysis over Arabic and Chinese by Ahmad et al. [2]. Their sentiment analysis framework consists of extracting financial terms using statistical models, and then they build a local grammar that is associated with each term using the term's popular collocations. Their hypothesis is that, in each domain, every term can be mapped to a limited list of local grammars that express sentiments. The technique was evaluated over a corpus of financial news articles gathered from Reuters written in modern standard Arabic.

Abbasi et al. [3] provide a valuable comparison between linguistic features selection variations between English and Arabic. In their work, they proposed the usage of entropy weighted genetic algorithm over a set of features that have been extracted from Arabic text to select the feature that best classifies the text according to its sentiment. After finding the appropriate set of features, the system uses SVM classifier to classify the Arabic text.

Additionally, Elhawary and Elfeky proposed an approach to mine Arabic business reviews using a straightforward lexicon-based technique [4]. In this approach, the authors propose to dynamically build a sentiment lexicon by starting with a set of seed words and expand the set by propagating through the Arabic similarity graph which clusters synonyms and antonyms. The resulting lexicon is then used to conduct sentiment analysis over a set of business reviews.

Last but not least is the effort of Abdul-Mageed et al. [5] to conduct sentiment in addition to subjectivity analysis to modern standard Arabic (MSA). In this research, they have opted to manually annotate a corpus of newspaper articles using the Penn Arabic Treebank which constructs a new Arabic polarity lexicon. Then a binary classifier classifies subjective and objective text. Afterwards, a second tier of classification is conducted using SVM classifier to detect negative and positive sentences.

## 4. System Design and Implementation

In our system, we aim to introduce a technique to solve the problem of identifying the overall sentiment contained in an Arabic piece of text generated by users and, consequently, detect the sentiment of the evaluative Arabic text found in Arabic websites and social networks that is pertaining to a specific topic or item.

To put our system into context and to facilitate the design and implementation of the system, we conducted our design and testing over the restaurant reviews domain. In addition to the vast popularity of restaurant reviews which appeal to a wide range of readers, we have chosen this domain due to the availability of a website called http://www.qaym.com/ which is rich with Arabic user reviews. Additionally, and most importantly, http://www.qaym.com/ provides an application programming interface (API) that enables our game component to extract user reviews in an easy and efficient manner.

Our proposed solution is an unsupervised technique that conducts fine-grained sentiment analysis which operates on the sentence level. Fine-grained is well known to be more informative than coarse-grained sentiment analysis which analyzes whole documents at a time [6]. Additionally, our choice of fine-grained rather than coarse-grained better suits our intended input which is Arabic reviews and opinions which we have found to be short and concise in nature. Moreover, we have designed the solution so that it heavily relies on a dynamically changing set of lexicons. By enabling the lexicon to be dynamically updated, it can adapt to changes in the trends of expression used to convey evaluative meaning. Furthermore, these lexicons will enable us to evade the problem of trying to conduct natural language processing over informal Arabic which is prone to yielding low accuracy results.

**Figure 1:**
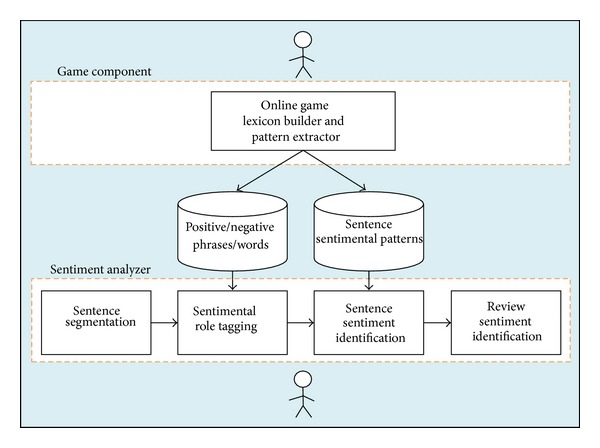
The architecture of our system depicting the game component and the sentiment analyzer.

Figure 1 illustrates the pipeline of our system including the used assistive components. Our system is divided into two separate components; the output of one is used as an assistive resource in the other. The first, and most straightforward, component is a free, online computer game that aims to collect significant annotations of reviews from online players by presenting the problem in a visually appealing and challenging context.

The other component, which is more significant to our problem, is the sentiment analyzer. The sentiment analyzer, by giving it a set of lexicons built using the previously explained game and the set of negative, positive, and neutral patterns, will classify user reviews according to their sentiments. The upcoming sections contain a detailed explanation of each component.

### 4.1. The Game Component

**Figure 2:**
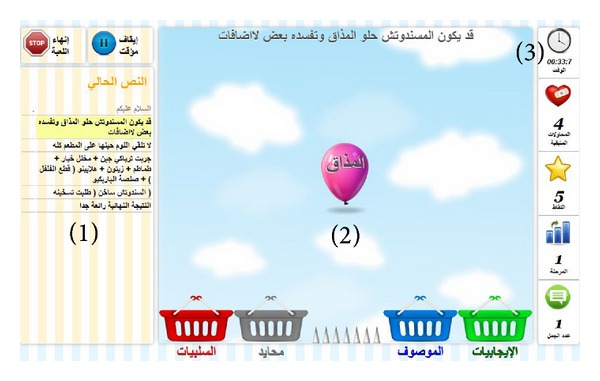
The interface of the game-based component. The interface consists of three panels. (1) The dashboard: it consists of control buttons that enable the user to pause the game or terminate it. It also consists of the review which is also the queue of the upcoming sentences in current game round. (2) The canvas panel: in it the words of the sentence will drop as balloons and the user saves the balloons from blowing up by dragging them to one of the four baskets. (3) The third panel is the statistics panel: it consists of basic statistics and information needed by the player to track his/her performance.

The game session consists of several rounds that will go on indefinitely. In each round, the game will retrieve a review from http://www.qaym.com/. The review will be broken into sentences. The review sentences will go one by one to the “sky” screen where each word in the sentence will fall in the shape of a balloon. The user is then required to study the context of this word in the sentence and then drag the balloon into one of four baskets. The “Negatives” basket indicates that this word in itself or as part of a whole phrase constitutes a negative phrase/word. The “Positive” means that the word constitutes a positive word/phrase. The “Entity” basket is for words that are being described in the sentenced (mainly nouns). Otherwise, for words not belonging to any of the previous baskets, the user may drag the balloon to the “Neutral” basket. However, if the user fails to drag the balloon, it will fall into the spikes and blow up. The user then loses a “life.” Losing 5 lives will terminate the game. The game will continue until the user exits the game or consumes the gamer lives.  Figure 2 illustrates the interface design of the online game.

After classifying each word individually, the user then must classify the whole sentence as negative, positive, or neutral, thus enabling the system to* extract the sentence pattern and its classification*. Each time the user classifies a word, the user will score a point. Classifying two reviews will advance the user to the next level which will increase the balloon falling speed to enhance the game challenge.

An important factor that must be taken into account is ensuring the best output possible of the proposed game. Such online game is easily subject to game cheaters or those who would insert any illogical solutions just to win a game. To ensure best results, games with a purpose usually incorporate antispamming measures by requiring two or more players to agree on the same result in order to take the result into account. This has been ensured in our game by adding a “frequency” counter. The counter will increment each time a positive/negative/neutral phrase receives the same classification by more than one player in different time. Thus, when the frequency counter is larger than one, the classification has gained consensus and is more reliable to be used.

To put the game players into context, the game was bundled in a website that briefly explains the purpose of the game. The game was then uploaded and made available through the World Wide Web (game URL: http://kalimat.afnan.ws/). The website content encourages visitors to enhance Arabic language research by playing the game as much as possible. It also encourages visitors to share the game using several online communication channels including social networking websites and email.

As the game continues to attract users, this will ensure the continuous update in the lexicons and will provide a cost-effective way of building rich informal Arabic lexicons of negative words, positive words, and domain-specific entities. The usage of these extracted resources will be explained in the next section.

### 4.2. Sentiment Analyzer Component

The sentiment analyzer is the main component of our system that specifies whether a review is considered negative or positive. In performing this task, the sentiment analyzer heavily relies on the resulting lexicons and sentiment patterns that were constructed using the game component.

The proposed architecture of our sentiment analyzer is a straightforward technique for lexicon-based sentiment analysis. It is thus designed to fully leverage the usage of our acquired lexicons. The aim is to cast greater focus on the novelty of the construction of these lexicons and employ them as an integral part of the system in order to be able to measure the success of the construction of these lexicons.

Firstly, the* sentence segmentation* subcomponent will break down the reviews into sentences which are the unit of operation of subsequent components. Secondly, for every word in the sentence, the* sentimental role tagging* identifies the word's role in the sentimental meaning of the sentence using the lexicons. The lexicons are acquired from our game component.

The third and final step is* sentence sentiment identification*. We have identified two different ways to achieve sentence classification using the sentiments of its words. First method is that, given the pattern of the words' role in the sentence (i.e., the word role and its location in relation to other words in the sentence), the pattern will be matched to a set of acquired annotated patterns that map to the sentence overall polarity. The other method is classifying the whole sentence according to the majority of sentimental words in the sentence. Each component is fully explained in the following subsections.

*Sentence Sentimental Patterns*. A set of annotated patterns are collected in the game component, where words are tagged according to their selected lexicon and the word is omitted if it does not belong to a lexicon. Then, each pattern is stored in the game database with its corresponding indicated polarity as a pair (*P*, SEN), where *P* is the pattern and SEN ∈ negative, positive, neutral, and sentiment (*P*) = SEN. In implementing the pattern matching approach, we identify function Pattern  (*S*) which takes the sentence *S* and returns its appropriate sentimental pattern that was derived from tagging the sentimental role of every phrase in the sentence:

(1) $$\begin{array}{c} P_{s}=P\left(S\right)=\left(t_{1},t_{2},\ldots ,t_{n}\right), \end{array}$$

where *t* can be “pos,” “neg,” “ent,” or “neu.” The algorithm, when identifying consecutive phrases of the same classification, will merge them into one tag in the pattern string. Furthermore, when the algorithm identifies a word to be a negation, it will negate the polarity of the phrase immediately next to the negation word; then the negation tag “NO” will be discarded.

**Figure 3:**
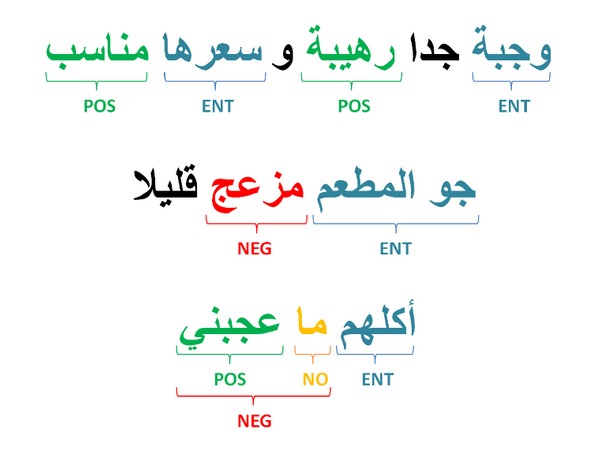
An example depicting a positive sentence and two negative sentences. In each sentence the significant words and/or phrases are tagged.

Figure 3 shows, in three different sentences, the significant words that influence the sentence polarity and their association with one of the three lexicons.

After extracting the pattern of the sentence, it is then looked up in the database to find its classification as per the game component output. We define a function Polarity  (*P*_*s*_) which returns the classifications of the pattern string *P*_*s*_ and the frequency of each pattern. The frequency signifies the number of times a sentence that has this pattern has been classified by a game player to be of this classification. So the function Polarity is defined as such:

(2) $$\begin{array}{c} \text{Polarity}\left(P_{s}\right)=\left\{\left({\text{class}}_{1},{\text{freq}}_{1}\right),\ldots ,\left({\text{class}}_{n},{\text{freq}}_{n}\right)\right\}, \end{array}$$

where

(3) $$\begin{array}{c} \text{class}\in \left\{\text{pos},\text{neg},\text{neu}\right\}. \end{array}$$

Finally, we deduce that the polarity of the sentence *S* is

(4) $$\begin{array}{c} \text{Polarity}\left(S\right)=\text{MAX}\left(\left({\text{class}}_{1},{\text{freq}}_{1}\right),\ldots ,\left({\text{class}}_{n},{\text{freq}}_{n}\right)\right). \end{array}$$

*Sentence Sentimental Majority*. It is another alternative approach to detect the polarity of each sentence. The system aggregates the polarities of each word or phrase in the sentence to output the overall polarity of the sentence. We call it the sentiment majority technique. It consists of two subapproaches: (1) majority approach with entity identification and (2) majority approach without entity identification.

*(1) Majority Approach with Entity Identification*. This approach will classify the sentence according to the dominating sentiment of the phrases contained in the database. Another way to explain this is as follows: after identifying the entity, negative and positive phrases in the sentence, every identified entity will be given weight of zero, negative phrases are given weight of −1, and positive phrases are given a weight of 1. Thus, if sentence (*S*_*j*_) consists of *n* phrases the weight of phrase number *i*  (*P*_*i*_) is

(5) $$\begin{array}{c} W\left(P_{i}\right)=\{\begin{array}{cc} 0 & \text{if}\;\text{polarity}\left(\text{phrase}\right)="\text{entity}"\\ 1 & \text{if}\;\text{polarity}\left(\text{phrase}\right)="\text{positive}"\\ -1 & \text{if}\;\text{polarity}\left(\text{phrase}\right)="\text{negative}". \end{array} \end{array}$$

Then, we define the weight of whole sentence *S*_*j*_:

(6) $$\begin{array}{c} W\left(S_{j}\right)=\overset{n}{\underset{i=1}{\sum }}W\left(P_{i}\right). \end{array}$$

Finally, we define the sentiment polarity of the whole sentence *S*_*j*_ to be

(7) $$\begin{array}{c} \text{Polarity}\left(S_{j}\right)=\{\begin{array}{cc} \text{Neutral} & \text{if}\;W\left(S_{j}\right)=0\\ \text{Positive} & \text{if}\;W\left(S_{j}\right)>0\\ \text{Negative} & \text{if}\;W\left(S_{j}\right)<0. \end{array} \end{array}$$

*(2) Majority Approach without Entity Identification.* Another variation of the previous approach suggests overlooking the entity identification in the sentence. As observed earlier, the entity phrase gives a weight of zero which does not affect the overall weight of the sentence. However, discarding the identification of entities gives slight changes in the algorithm performance. This is due to the fact that there are some collisions in the database. Some phrases might be enrolled as entities and as negative or positive phrases at the same time. The algorithm is programmed that whenever such collision is detected, it will take the one with the highest classification frequency. So, in the first variation of the algorithm, the entities will be identified as such if they have high frequency which will prevent the algorithm form identifying the phrase as negative or positive if a collision exists. In the second variation, the algorithm will not look into the possibility of the phrase being an entity and will classify it as a positive or negative phrase even if a collision exists and the phrase has higher frequency of being an entity.

## 5. System Evaluation

The task of sentiment analysis is generally regarded as an information retrieval (IR) problem. IR problems are the set of problems concerned with searching for related documents, information within documents, or metadata that can be deduced from these documents.

Systems that fit into the criteria of IR problem can have their performance measured using specific agreed-upon metrics. These metrics are as follows [7].

*Precision.* This measure indicates the “repeatability” of the measurement. This means that when the system tries to resolve the problem under the same conditions, precision indicates how close the results are to each other. Calculating precision is done by dividing the number of correctly classified as positive results by the number of overall results that were classified as positive whether the classification was correct or not. The precision is then calculated as follows:

(8) Precision=Number  of  correctly  classified  opinions×(Number  of  correctly  classified  opinions+number  of  falsely  classified  opinions)−1.

*Recall.* This measurement indicates the percentage of the completeness of the result. Low recall would mean that there are many left-out results that were not classified. Recall is calculated by dividing the number of correctly classified results as positive by the number of all positive correct results that should be classified as positive. Recall will be measured as follows:

(9) Recall=Number  of  correctly  classified  opinions×(Number  of  correctly  classified  opinions+number  of  opinions  that  were  not  classified)−1.

When calculating the precision and recall of our proposed system, we will benchmark the data using a dataset that has been annotated by a human expert (Arabic linguist).

In order to conduct system evaluation, we have acquired a test set that consists of 5000 sentences that have been deduced from restaurant reviews. Then, these 5000 sentences were classified as positive, negative, or neutral according to their sentiments by a human linguist. Then, we have employed our sentiment classifier to produce the sentiment classification as explained previously.

To calculate the accuracy of our system we have compared the annotations of the test set with our system outcome. As previously explained in the system implementation part, the sentiment analyzer runs three major versions of the classification algorithm:* pattern matching approach, majority approach with entities, *and* majority approach without taking entities into account*. The difference between the two last approaches is that disregarding entities will give high priority to phrases that have been misclassified as entities.

The pattern variation of our algorithm will detect all the entities, positive, negative, and neutral phrases, and then extract the pattern of the sentence; if a phrase is found to have different classifications, the priority is given to the classification that has the higher frequency. Afterwards, the algorithm will look up the exact pattern from the patterns database which maps to its overall sentiment. In our system this algorithm variation, when applied to classify the 5000 test set sentences, reached a precision level of 56.14%. The system was able to find 3109 matched patterns. Furthermore, it has reached a recall level of 59%. The recall measurement indicates that 59% of patterns were found in the database and were mapped accordingly; the remaining patterns that did not have a previous entry in the database were classified according to their dominant sentiment (majority algorithm).

The* second* variation of the algorithm conducted a majority classification. This classification will detect entities, positive and negative phrases in the algorithm. Then assign a weight of zero for each entity, 1 for positive phrases, and −1 for negative ones. If the summation of all weights in the sentence is less than zero, the sentence is considered as negative; if it is more than zero, the sentence is classified as positive; finally, if the overall weight is zero then the sentence is considered to be neutral. This classification algorithm yielded a better classification performance as it reached the accuracy of 60.5%. This is higher than using sentiment patterns to classify sentences. Additionally, as the algorithm indicates, the recall for this variation is not possible to calculate as all sentences will be classified.

**Figure 4:**
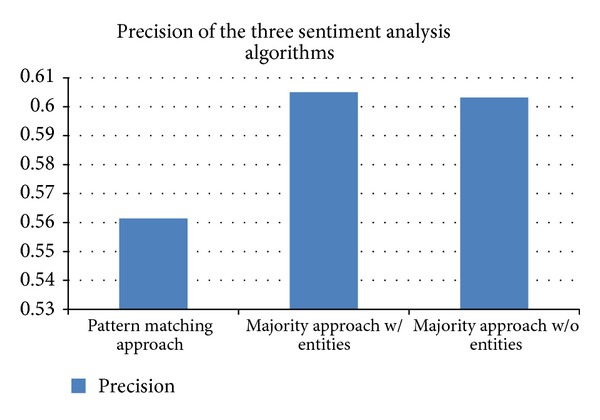
Benchmarking the precision results of the three different variations of the classification algorithms.

The* third* variation of the classification algorithm specifies that, since the entity plays no role in tipping the overall weight of the sentence, it should not be detected nor identified. This poses a difference to the algorithm performance. If entities were taken into account, then if a phrase is detected to have been classified by players both as a polar phrase and as an entity, the higher frequency of the two has the higher priority. Consequently, the phrase polarity will be discarded. However, in this variation of the algorithm, the entities will not be detected, giving the polar phrases higher priority even if the same phrase has been identified as an entity. This variation yielded a precision of 60.32%, giving slightly less accuracy than the previous variation. The different precision results of all three variations are benchmarked in Figure 4.

## 6. Discussion and Limitations

As the previous section explained, our system has three different approaches to employ its linguistic resources to perform sentimental classification. The main approach which employs extracting the pattern of sentimental tags in the sentence yielded a relatively low accuracy result of 56.14%. When observing the reasons behind this failure, we observed two cases: the extracted pattern is either too common or too obscure and specific.  Table 1 shows examples of these cases in the output of this algorithm. For the first case, the pattern is actually enlisted to have all three polarities. Although our algorithm takes the one with the highest frequency, this is not always accurate. Since this exact same pattern was classified to have another polarity, it must be true for some cases even if its frequency is not the highest. A solution should be developed to add another heuristic that guides the selection of the appropriate pattern classification other than the frequency.

The second algorithm classifies the sentence according to the classification of the maximum number of its phrases. That is, if the sentence has more positive phrases than negative ones, it will be classified as positive. This algorithm succeeded to classify more than 60% of the cases. The first variation was implemented to take into account the classification of entities. This poses a difference since the algorithm, when identifying phrases in the sentence, will look up the phrases in the database; then when a phrase has more than one classification (i.e., was classified as both entity and positive or negative phrase), the algorithm will give priority to the classification that has the highest frequency. So, as it was expected, although identifying entities in itself does not affect the overall weight of the sentence polarity, it prevents the classification of some phrases as negative or positive phrases when they have been identified as entities. Hence, the majority with entities variation of the algorithm scored an accuracy of 60.5%, while without the identification of entities, it scored 60.32%; the best accuracy result reached by our system is 60.5%. Although this percentage fails to impress, it is slightly higher than some Arabic classification algorithms that have been discussed earlier. An argument can be made that, in fact, comparing our system results to previous approaches conducted over sentimentally analyzing Arabic text would not yield fair comparisons due to two factors.

- Our sentiment analyzer conducts sentiment classification over informal Arabic which was not attempted by previous Arabic sentiment analysis endeavors. Informal colloquial Arabic is generally characterized to be highly ill-structured, inconsistent, and difficult to process.
- The training of our system was intended to be conducted over more than 10000 sentences. However, since the training is done using an online game component which was deployed for a limited amount of time and yielded less than average player base, it resulted in the classification of only 2600 sentences. When taking into account that a sentence must be classified twice to get more accurate results, our system did not reach its full potential. Regardless of the limited training, we have conducted the testing over 5000 words (twice the amount of the training set) and yielded an accuracy of 60.5%.

When taking these two factors into account, the accuracy seems very impressive considering these limitations. We predict that even higher accuracy had the game component succeed to gather more data. The failure of the game component to produce more entries can be attributed to two factors.

First is the time during which the game is deployed online. In our system the game has been uploaded and targeted by a visitor for approximately a month. During this month only 24% of the training set was classified. We can argue that, as the game gains more popularity as time passes, it would generate a large number of outcomes.

Secondly, the game components themselves fail to retain a large number of players. Several game testers expressed their boredom during gameplay; this is attributed to the fact that the purpose of the game prevents it from awarding the player according to their performance. That is to say, whichever the classification the player chooses for the phrase, it counts as a point, without providing feedback whether the classification is correct or not. Generally, this is due to the great difficulty at which anyone can convert the problem at hand to an enjoyable linguistic game. Game logistics and environment that make the user do some phrase sentiment classification that is not known by the game in a fun and compelling way are very hard to design. A suggestion to enhance the game outcome is by deploying it using several gaming consoles in addition to the online interface. Furthermore, the data collection can be conducted using several other outlets other than a game.

## 7. Conclusion

In this paper we have proposed a novel technique to solve the problem of sentiment analysis of colloquial Arabic found in online evaluative websites [8]. We used human computation approaches to design an online game that produces a set of valuable resources used in our sentiment analyzer.

The system performed fairly as it was able to classify 60.5% of the sentences correctly. Major limitations hindered the enhancements of this result, one of which is the inadequacy of the game component to attract more players during the short period it was deployed. We suggest improving the game enjoyment and increasing the number of outlets that can produce the game lexicons. Such outlets include providing the problem of sentiment annotation of text as a CAPTCHA solution.

## Figures and Tables

**Table 1:** Sample output of the pattern matching approach.

Sentence	Pattern	Correct classification	Found pattern classification
للأسف كنت أقدر هالمطعم	neg, neu, ent	Negative	neg (freq = 3) pos (freq = 1)

وأسعارهم مناسبة	ent, pos	Positive	pos (freq = 90) neu (freq = 2) neg (freq = 1)

الأسعار عادية	ent, neu	Positive	neu (freq = 34) pos (freq = 25) neg (freq = 2)

المطعم جيّد لكنه عادي ولا شيء يميزه	ent, pos, neu, neg, neu, pos	Neutral	(Not found)
